# Seismic Behavior of Concrete Columns Retrofitted with a Brace-Type Replaceable Steel Link

**DOI:** 10.3390/ma16031182

**Published:** 2023-01-30

**Authors:** Min Sook Kim, Young Hak Lee

**Affiliations:** Department of Architectural Engineering, Kyung Hee University, Deogyeong-Daero 1732, Yongin 17104, Republic of Korea

**Keywords:** seismic retrofit, brace, replaceable, energy dissipation, cyclic loading test

## Abstract

This paper presents the results of a combined cyclic loading test on a single reinforced concrete column which was retrofitted with a newly proposed brace-type replaceable steel link. A total of four retrofitted reinforced concrete columns, with the length of the brace as a variable, were fabricated and tested. A companion column without retrofitting was used as the control specimen. The test results indicate that the proposed brace-type replaceable steel link can be effective in retrofitting the concrete columns, resulting in improvements in the strength, stiffness, and energy dissipation of columns. We observed that the maximum load increases by at least 87%, effective stiffness increases by 44%, and energy dissipation capacity increases by 91% when compared with non-retrofitted specimen.

## 1. Introduction

The use of a brace is preferred for the seismic retrofit of reinforced concrete frames due to its high strength-to-weight ratio, simplicity in application with minimal demolishing, rapid construction, and its ability to enhance the global seismic resistance of the structure [1,2]. The efficacy of braces in dissipating the input of seismic energy largely depends on the design and detailing of the gusset plates connecting the braces with the beams and columns. The brace stress is transferred to the concrete frames by the gusset plates connecting the existing structures and the braces [3,4]. Particularly with double K-braced frames, significant stresses and deformations may occur since two braces are joined to one gusset plate, and complicated analysis and design are needed. When retrofitting reinforced concrete frames with braces, the connection between the ends of the braces and the concrete frame is a key challenge. Therefore, many studies have been conducted to investigate the brace–concrete frame connections. Zhang et al. [5] conducted experiments on 12 specimens to study the low-cycle fatigue behavior of I-shaped steel bracing members with gusset plate connections, and the results showed that there were three locations vulnerable to damage following three different damage sequences. Ebrahimi et al. [6] confirmed the gusset plate played an important role when transferring stresses, so should be considered according to the details of the columns. Ebrahimi et al. [6] noted that limited studies have been carried out on the gusset plate to hollow structural section (HSS) column, and proposed a new gusset plate connection passed through HSS column called the through-gusset plate. Dong and Wang [7] developed a new type of joint structure with greater ductility and higher strength called beam–column joint with a gusset plate angle (JGA), and the influence of each of the joint components on the overall performance of the structure was evaluated by finite element analysis and experiment. Mirhosseini and Hamzeh [8] proposed a Bar–Fuse damper (BFD) model to prevent brace buckling and increase the ductility of the concentrically braced frame (CBF) along the longitudinal axis of the diagonal brace to the gusset plate, and proposed a design procedure. The structural members resist seismic loads, the joint of concrete column and beam tend to either open or close due to the deformation of the structural members. Therefore, the brace–concrete frame connection is subjected to additional tension or compressive forces that may lead to deformation or buckling of the gusset plates. The above literature review on gusset plates shows that, although quite a few studies have been carried out to address behavior and analysis of gusset plates, experimental research on improving the connection details of the brace is limited.

The brace may be a useful seismic retrofit device despite the problem of the gusset plate. However, the buckling that occurs in conventional braces causes a sharp decrease in load-resisting and energy dissipation capacity. Buckling-restrained brace (BRB) is proposed by many researchers. Saingam et al. [9] pointed out the design method of retrofit using the equivalent linearization approach does not consider the additional stiffness due to the composite behavior between the RC frame and the elastic steel frame. They proposed a numerical model incorporating the composite behavior and calibrated against quasi-static cyclic loading tests. The analysis results suggest the proposed retrofit design method can more accurately estimate the lateral stiffness of the retrofitted structure in order to consider composite behavior. Sitler et al. [10] proposed new multistage BRBs which have a similar composition to conventional BRBs, and can employ a similar fabrication and design method. The multistage BRBs consisted of low yield point (LYP) and high yield point (HYP) cores, and provide multistage behavior. In moderate earthquakes, the LYP core yields and dissipates energy and the HYP core provides elastic restoring force, reducing residual drifts. At large drifts, the HYP core yields and the device behaves as a conventional BRB. The device behavior and multistage response were evaluated by a cyclic displacement-controlled test. The experimental results confirmed the feasibility of the multistage BRBs configuration and multistage behavior. Zhao et al. [11] proposed a rapid seismic retrofitting method using a gapped eccentric steel brace (GESB) system for the rehabilitation of damaged RC frames. The GESB system has a parabolic metallic damper which can provide a rapid retrofitting scheme. The pseudo-static cyclic loading test was conducted to evaluate the seismic performance of the GESB system. The test results confirmed that the proposed GESB system eliminated any local repair or grouting, and thus allowed the rapid recovery of structural performance. The energy dissipation capacity of the retrofitted frame increased significantly faster than the non-retrofitted frame.

The repair or replacement of primary structural members that have experienced an earthquake is uneconomical and complicated to construct. Therefore, an alternative approach is to concentrate damage on a structural element, such as braces, that are easy to repair or replace. In this paper, a brace-type retrofitting method is proposed by introducing a replaceable steel link (RSL). This new RSL consists of a single replaceable steel link that acts as brace, and two steel plates with welded hinges: one attached to a column; and the other to a beam or a slab using chemical anchors. The replaceable steel link to the hinge of each plate is fastened with bolts. The steel link installed in the sliding slot hinge can move horizontally along the lateral displacement of the column through the sliding slot. The deformation or failure of the brace is the preferred failure mode rather than the secondary failure modes, such as the failure of the gusset plates. The key advantage of this system is that it consists of replaceable brace and hinges that can absorb deformation. In addition, it has very simple details compared to the conventional brace systems. A total of four retrofitted RC columns and one control column specimen were fabricated and tested to evaluate whether the proposed retrofitting details were capable of improving the load-carrying capacity and ductility of the concrete columns.

## 2. Seismic Retrofit Method Using Replaceable Steel Link System

**Figure 1:**
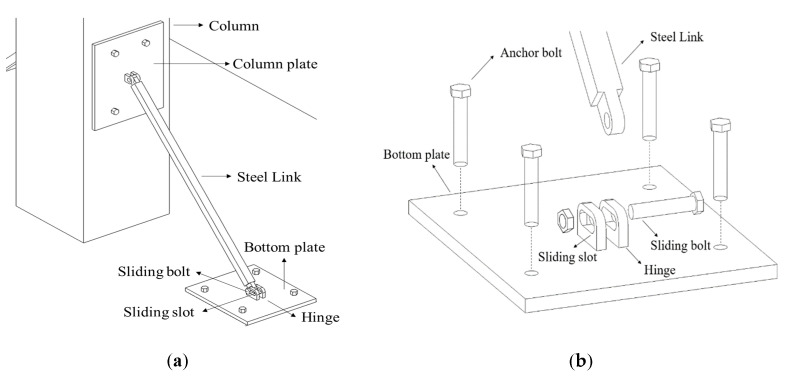
Scheme of the replaceable steel link (RSL) system: (**a**) applications of RSL; (**b**) components of the RSL system.

Unlike conventional braces, the RSL system does not have a gusset plate with complicated details, and it can be diagonally connected between a column and a beam, or a column and a slab. The retrofitting scheme of the proposed system is shown in Figure 1. The RSL system consists of one steel link and two steel plates for attaching the steel link to the column and beam or slab. The steel plate attached to the column is fixed by steel links with bolts. The steel plate attached to the beam or slab is welded to a hinge with a slot, and the steel link and hinge are fastened by a sliding bolt. The steel link dissipates energy as the bolt in the sliding slot moves with frictional force due to the lateral displacement occurring in the column. When the lateral displacement of the column increases, the bolt can no longer move, and from this point the steel link behaves like a brace. Buckling or deformation in the steel link is induced before the longitudinal reinforcement of the column. 

The steel link can absorb the deformation energy by moving along the displacement of the column through the sliding slot, creating friction between the sliding bolt and the slot. The capacity for the stiffness and deformation of the column can be set by adjusting the allowable range of movement of the sliding bolt in hinge. The position of the plate attached to the column and beam or slab, and the link length according to the desired retrofit length, can be adjusted. Even if the steel link is buckled or failed in an earthquake, it can be replaced with a new one for the next earthquake. An additional advantage of the RSL system is that a single column can be selectively retrofitted. Seismic retrofit performance can be enhanced even if applied only to the plastic hinge region in column, which can be constructed in little space and causes no inconvenience in using the building during and after the construction.

## 3. Experimental Program

### 3.1. Specimen Details

**Figure 2:**
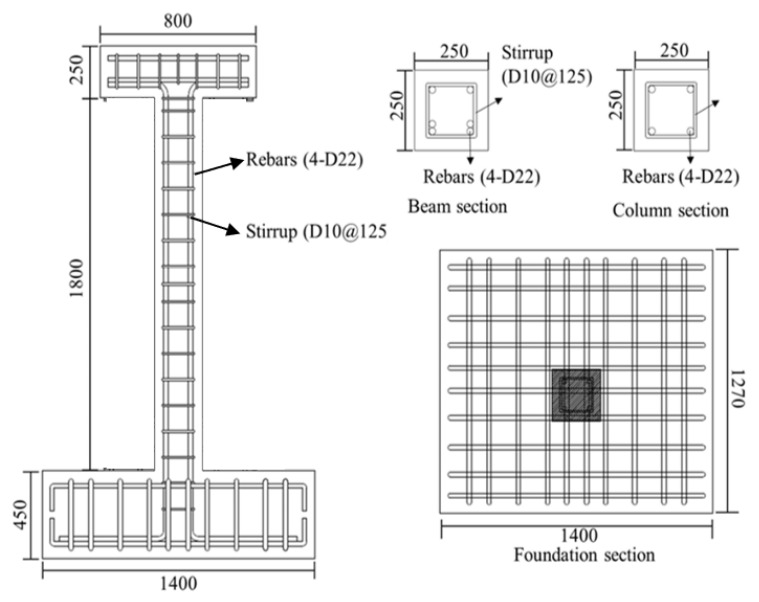
Details of an RC column (m).

Five reinforced concrete columns, including one non-retrofitted column and four columns with two types retrofitting length, were fabricated. The lengths of the retrofitting schemes, featuring two different plastic hinge lengths, were determined to be 375 mm and 575 mm according to Equations (1) and (2). For the reliability of the experimental data, two specimens with a retrofitting length of 375 mm and two with a retrofitting length of 575 mm were fabricated. The plastic hinge length was calculated by the equation from Panagiotakos and Fardis [12]:

(1) $$L_{p}=0.08L+0.022f_{y}d_{b}\geq 0.044f_{y}d_{b}$$

(2) $$L_{p}=0.18L+0.021d_{b}f_{y}$$

where *L* is the column length, $f_{y}$ is the yield strength of the longitudinal reinforcement, and $d_{b}$ is the diameter of the longitudinal rebar. The types and variables of the specimens are shown in Table 1, and the details of the specimens are shown in Table 2. Figure 2 shows the geometry and reinforcement details for the specimens. All specimens have a square column with a height of 1.8 m and a cross section of $250\times 250{\;\mathrm{mm}}^{2}$. An upper beam for loading and a foundation for fixing the specimen were manufactured. The upper beam also had a cross section of 250 mm×250 mm and a length of 800 mm, and the foundation had a cross section of 1400 mm×1270 mm and a depth of 425 mm. The diameter of the longitudinal rebar and transverse rebar were 22 mm and 10 mm, respectively, for the column, upper beam and foundation. All specimens on which the experiment was performed were reinforced with stirrup with a 90° hook to apply non-seismically details. The concrete used was 24 MPa and all specimens were allowed to cure for more than 28 days before testing.

**Figure 3:**
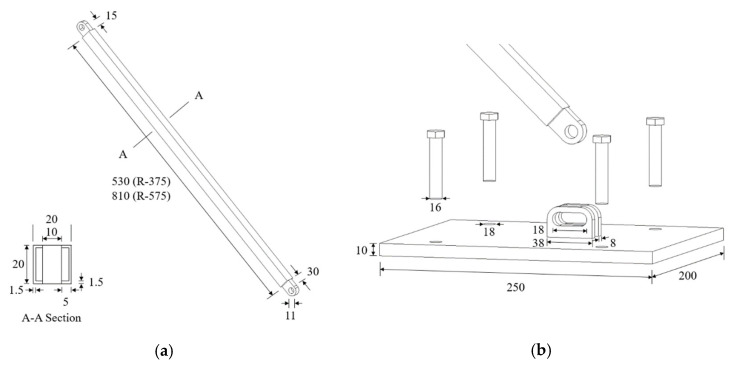
Dimensions of the RSL system (mm): (**a**) Steel link; (**b**) Bottom plate.

Two RSLs were installed on the left and right sides of the column. The RSL systems were attached using a total of eight anchor bolts for each system, each with an insertion length of at least 100 mm. Four anchor bolts with a diameter of 16 mm and an effective length of 100 mm were constructed. The pulling strength of anchor bolts was checked with the Hilti PROFIS Engineering Program [13] and developed based on ACI 318-19 [14]. Steel with a yield strength of 275 MPa was employed for the RSL system. The dimensions of RSL system are shown in Figure 3. 

### 3.2. Test Setup

**Figure 4:**
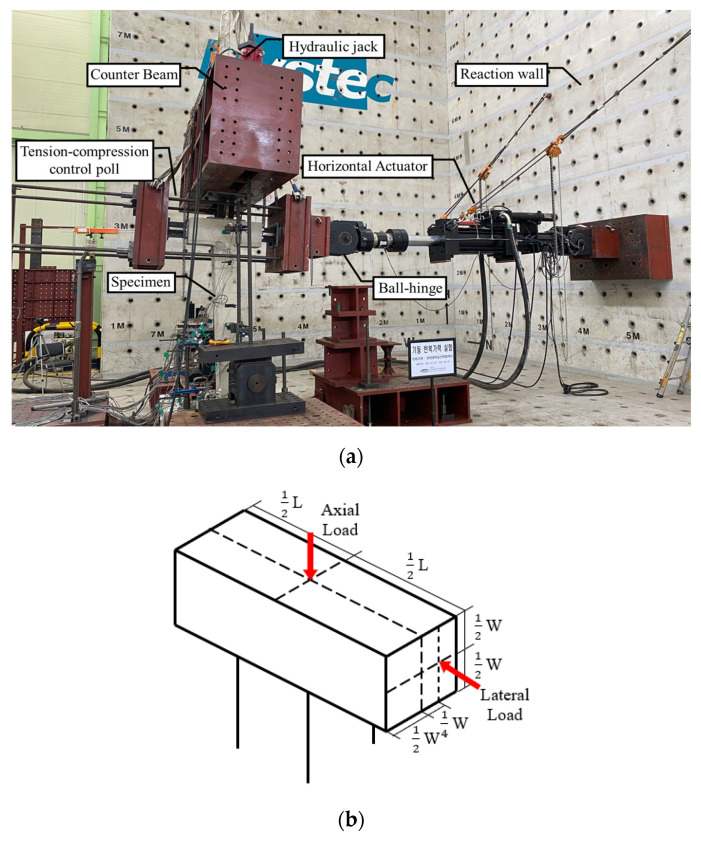
Experimental setup for the combined cyclic loading test: (**a**) Test setup; (**b**) Loading method.

The cyclic loading test setup is presented in Figure 4. The columns are subjected to combined loading from an axial force, including bending and a torsion moment during seismic excitations due to the multi-directional characteristics of earthquakes. The behavior of columns subjected to combined loading should be considered as essential for evaluating the seismic performance. In this paper, cyclic loading was applied to the upper beam with eccentricity of 65 mm to consider torsion, using a hydraulic actuator with a maximum load of 1000 kN as shown in Figure 4b. The actuator was installed between the reaction wall and the test specimen. The upper beam and the actuator were totally fixed to apply lateral load in both directions. The constant axial load was 255 kN, which is 17% of the axal load capacity of the column. 

**Figure 5:**
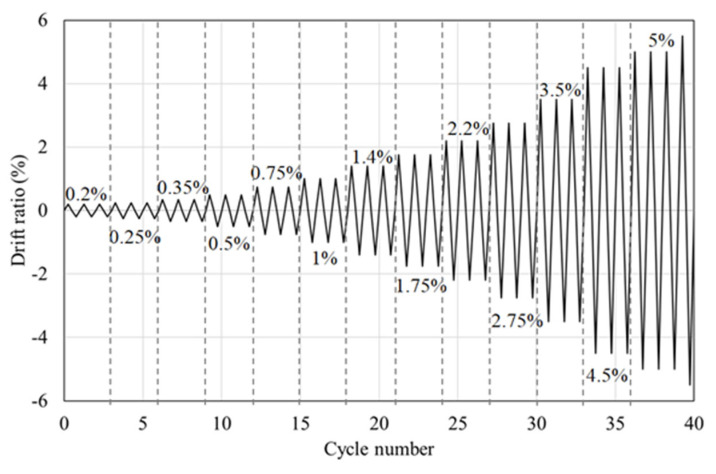
Loading Protocol.

The foundation was fixed to the floor by six anchor bolts to prevent any interference with the experiment results. The loading method was referenced to the load protocol given in ACI 374.1 [15], which states that the initial drift ratio should be within a range that confirms the linear elastic behavior, the subsequent drift ratio should not exceed 0.25%, and the subsequent step should be neither too large nor too small. As shown in Figure 5, the load protocol was set to be cycled three times for each drift ratio, followed by an incremental increase to 105 mm; this corresponded to a maximum drift ratio of 6%.

**Figure 6:**
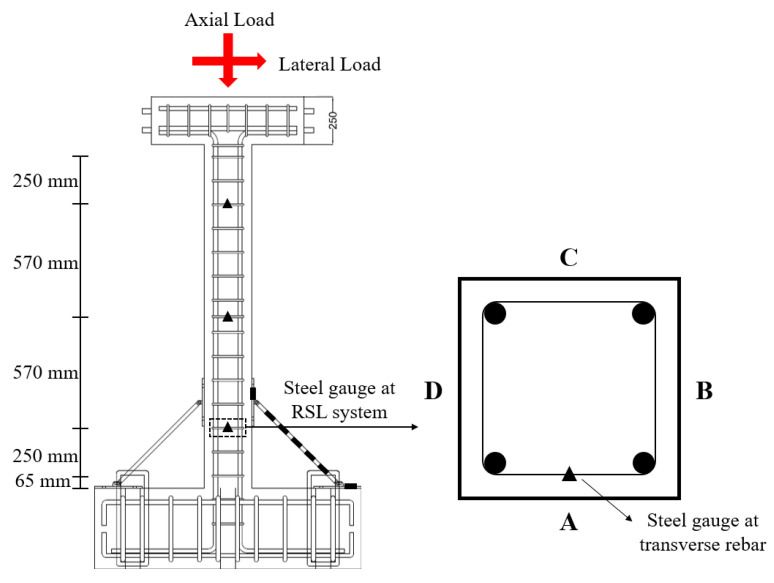
Locations of strain gauges.

Each retrofitted column was instrumented with 10 strain gauges attached to the transverse rebars, and RSL system. Transverse rebar strain gauges were placed at 65 mm, 875 mm, and 1685 mm above the foundation, and they were attached on only one side. RSL system strain gauges are attached one on each steel plate and five on the steel link. The locations of the strain gauges and names of the sides are shown in Figure 6. The surface of the specimen was named in alphabetical order in the counterclockwise direction, starting with A from the surface where the actuator was installed.

## 4. Experimental Results and Analysis

### 4.1. Cracks and Failure Modes of Specimens

**Figure 7:**
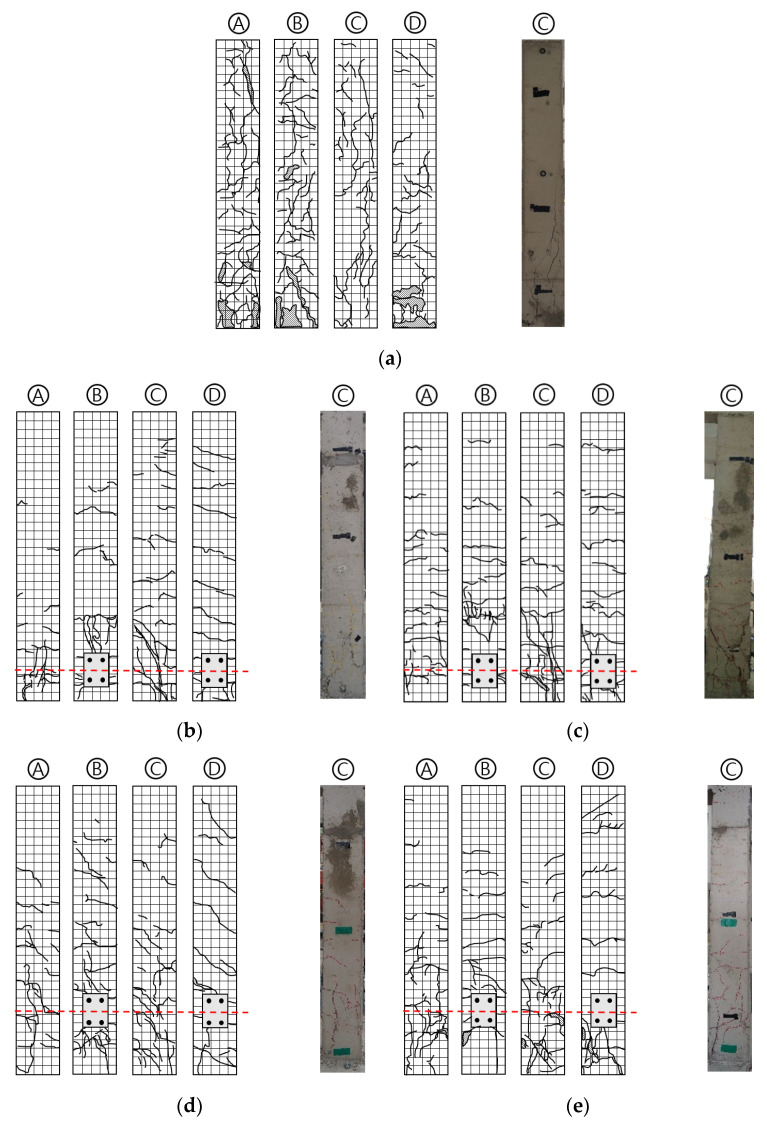
Crack patterns and failure of the specimens: (**a**) NR; (**b**) R-375-1; (**c**) R-375-2; (**d**) R-575-1; (**e**) R-575-2.

The crack patterns and C sides of each specimen after the experiment are shown in Figure 7. At the drift ratio of 0.75% for all specimens, initial flexural cracks appeared evenly on the middle of the C side of the actuator. All specimens started with the occurrence of initial flexural cracks, and the flexural cracks gradually spread widely as the load increased. After that, as shear cracks occurred, the spalling of the concrete became severe and the experiment was terminated. For the NR specimen (the non-retrofitted RC column), flexural cracks began to occur in earnest at the drift ratio of 1%, and shear cracks at the drift ratio of 1.4% as the lateral load increased. As the drift ratio increased, the flexural cracks widened, resulting in the occurrence of diagonal tension cracks between the existing cracks; then destruction and spalling of concrete occurred, followed by shear failure. At the drift ratio of 2.75%, the experiment was terminated at the drift ratio of 4.5% due to destruction of the bottom of the column, when the lateral load fell below 85% of the maximum load.

In the case of R-375-1, shear cracks initially occurred at the drift ratio of 2.2%. The width of the shear cracks increased significantly from the drift ratio of 4.5%, the concrete began to spall, and the bottom of the specimen was severely spalled at drift ratio of 5% and was finally failed. The behavior of R-375-2 was similar to that of R-375-1 because they had equal details, and the shear cracks initially occurred at the drift ratio of 2.2%. The R-375-2 specimen was failed at the drift ratio of 5%. The R-375 specimens had a greater drift ratio of crack occurrence than the NR specimen. The R-575 specimens incurred the initial shear crack at the drift ratio of 2.75%, which was larger than NR and R-375 specimens. The performance of suppressing the occurrence of shear cracks was better for R-575 specimens than for R-375 specimens. The experiments on both R-575 specimens were terminated at drift ratio of 4.5%.

**Figure 8:**
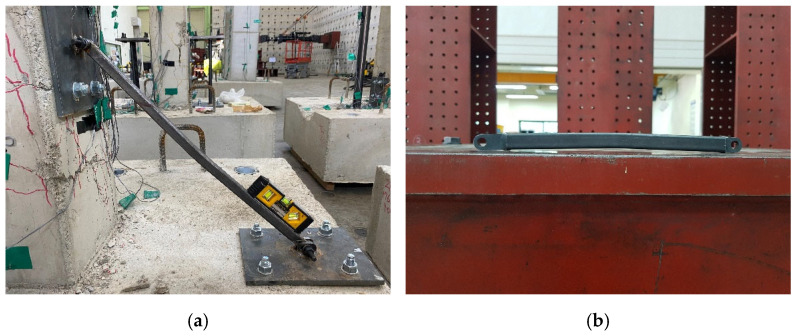
The buckled steel links: (**a**) Buckled steel link of R-575 specimen; (**b**) Buckled steel link of R-375 specimen after detaching.

**Figure 9:**
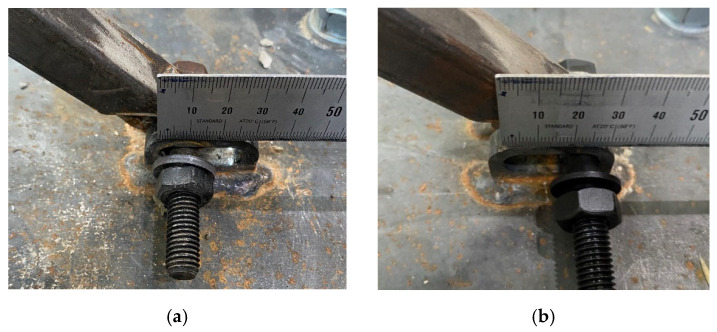
The sliding effect of RSL system: (**a**) Hinge before applying lateral load; (**b**) Sliding bolt reaching the end of the sliding slot.

The main differences between the non-retrofitted specimen and retrofitted specimens were the frequency of occurrence of flexural cracks and the location of shear cracks. With the NR specimen, the entire surface was covered with flexural cracks and shear cracks, and concrete spalling occurred severely at the plastic hinge region. However, in the case of retrofitted specimens, only flexural cracks often occurred only in upper end, and shear cracks did not occur much. Comparing the retrofitted specimens with different retrofit length, cracks occurred more intensively at the height at which the plate was fixed to the column. Since the anchor bolt is inserted through the column of more than 100 mm, cracks can easily occur. Therefore, cracks in the R-575 specimens occurred higher in the lower part of the column than in the R-375 specimens. Shear crack occurred at a smaller drift ratio in R-375 than R-575, but the final failure of R-575 occurred earlier than R-375. There was no bolt loosening in any of the specimens, which means the plates anchored to the column and foundation were not damaged, and the steel link had the desired retrofit effect, absorbing all the stress properly. The buckled steel link is shown in Figure 8, and the sliding effect of the RSL system is shown in Figure 9.

### 4.2. Load-Displacement Relationships

**Figure 10:**
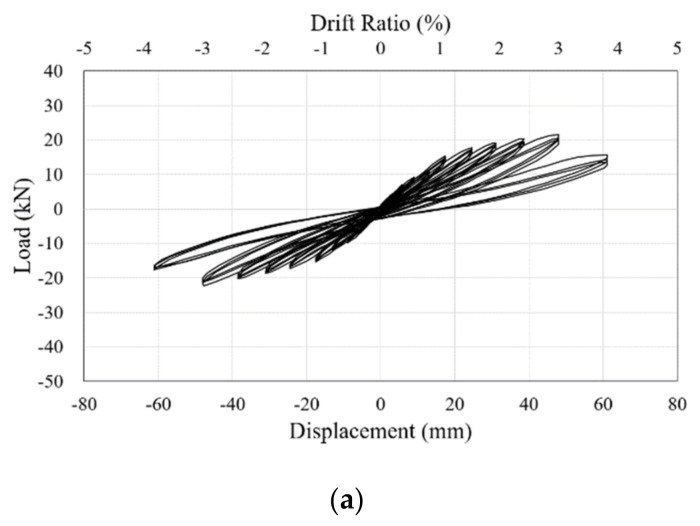

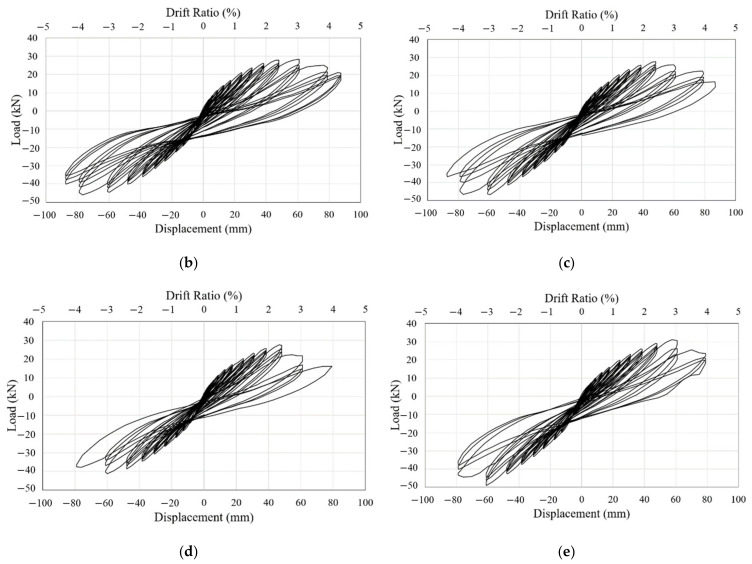
Load-displacement curves of the specimens: (**a**) NR; (**b**) R-375-1; (**c**) R-375-2; (**d**) R-575-1; (**e**) R-575-2.

**Figure 11:**
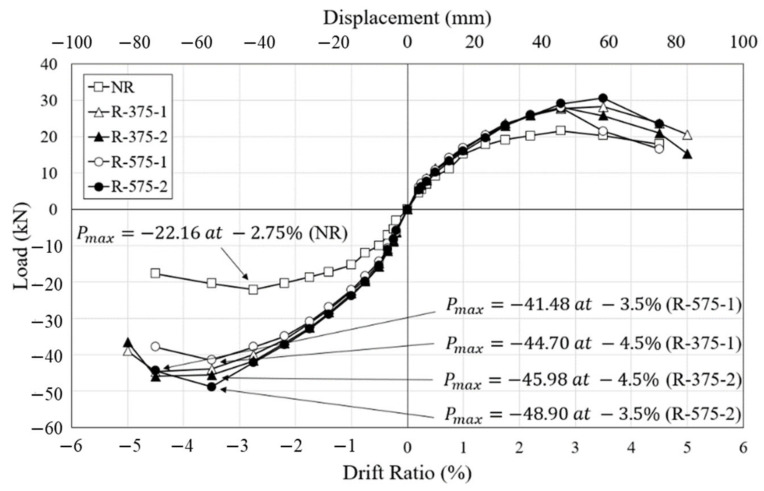
Envelope curves.

The hysteretic behaviors of each specimen according to the cyclic lateral load are shown in Figure 10, along with the load-displacement curves for all of the specimens. All of the maximum lateral loads according to the drift ratio can be found in the envelope curves for the specimens, shown in Figure 11. Analysis of hysteretic curves is performed considering the energy dissipated during cycles, the secant stiffness degradation of subsequent cycles, and the degradation of strength between two consecutive cycles at the same drift level. In common with all the specimens, the lateral load gradually increased as the experiment progressed and then decreased after reaching the maximum load. When the maximum loads of the specimens were reduced to about 80% to 85%, the experiments were terminated due to failure of the lower part of the RC column. The energy dissipation curing cycles can be evaluated as the area of the hysteretic curve. It can be seen that the area of the specimen retrofitted with the RSL system is wider than that of the un-retrofitted, NR specimen. The degradation of secant stiffness and strength of the retrofitted specimens R-375 and R-575 was smaller than that of the NR specimen. The secant stiffness degradation is mainly affected by the cracking. It was demonstrated that the proposed retrofitting system is effective in crack control as well as energy dissipation. 

As shown Figure 11, the initial stiffness of all specimens was almost identical. It was demonstrated that retrofitting with the RSL system has little effect on the initial stiffness of the concrete column. The maximum lateral load of the NR specimen was 22.16 kN at the drift ratio of 2.75%, after which, due to widened shear cracks at the bottom of the column, the concrete spalled and the lateral load was reduced. The maximum load subsequently decreased to 17.64 kN, which was about 80% of the maximum load at the drift ratio of 4.5%. In the R-375 specimen, the maximum loads were reached at the drift ratio of 4.5%. The maximum lateral loads of R-375-1 and R-375-2 were 44.70 kN and 45.98 kN, respectively, which were about 2.02 times and 2.08 times the maximum load of the NR specimen. The maximum loads of R-375-1 and R-375-2 decreased to 35.55 kN and 36.54 kN, respectively, which were about 80% and 79% of the maximum load of the NR. R-375-1 nd R-375-2 were destroyed by severe shear cracks and spalling of concrete at the drift ratio of 5%. The maximum lateral loads of R-575-1 and R-575-2 were 41.48 kN and 48.90 kN, respectively, at the same drift ratio of 3.5%; these were about 1.87 times and 2.21 times the maximum load of the NR. In terms of load-carrying capacity, the specimens retrofitted with the RSL system show the upper bound on the backbone envelopes. The R-575-2 specimen had the highest lateral strength, followed by the R-375-2 specimen, and the NR specimen, respectively. The load-displacement response of the retrofitted column was considerably improved with the employed retrofitting techniques. When comparing the average maximum loads of R-375 and R-575, there is only a difference of 0.3%. Since there is little difference in these experimental results, the plastic hinge length was not an important factor in retrofitting the RC columns. 

### 4.3. Strains

**Figure 12:**
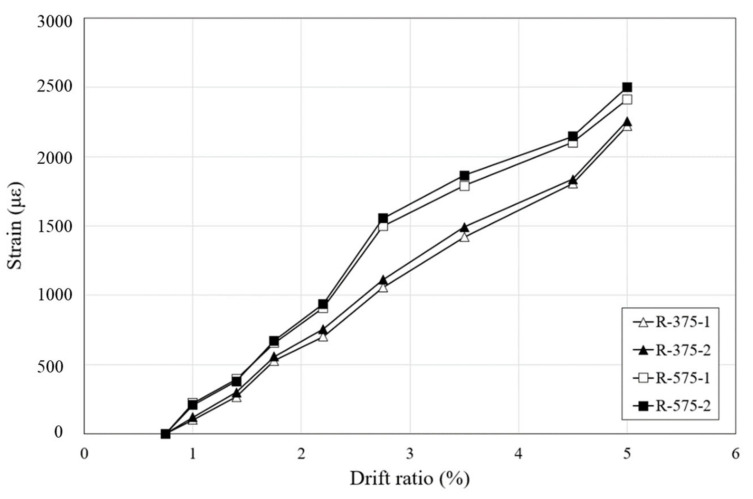
Strain-drift ratio curves of RSL system.

To check whether the RSL system effectively loaded the stress, strain gauges were attached to the steel link to measure the strain. The strain-drift ratio graph, shown in Figure 12, displays the strain measured at the center of the steel link, which loaded the greatest stress among the five locations. The RSL system installed on all specimens did not deform until the drift ratio of 0.75% was reached, and at the drift ratio of 1% the sliding bolt connected to the steel link reached the sliding slot. The steel link then began receiving load and measuring the strain value. As the lateral load increased, buckling and yielding were reached. Specimens with a retrofit length of 375 mm showed a similar pattern and yielded at a drift ratio of 2.75%, and specimens with a retrofit length of 575 mm yielded at a drift ratio of 2.2%. This is because the buckling load was smaller due to the larger slender ratio of longer steel link.

**Figure 13:**
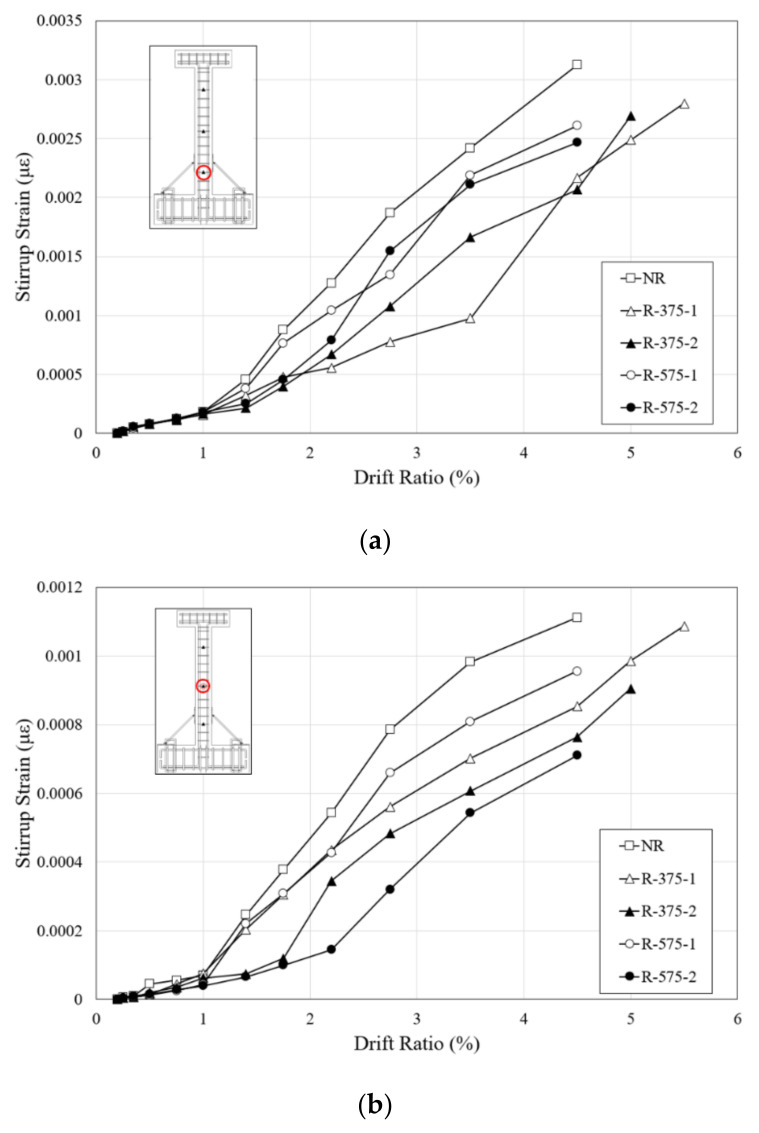

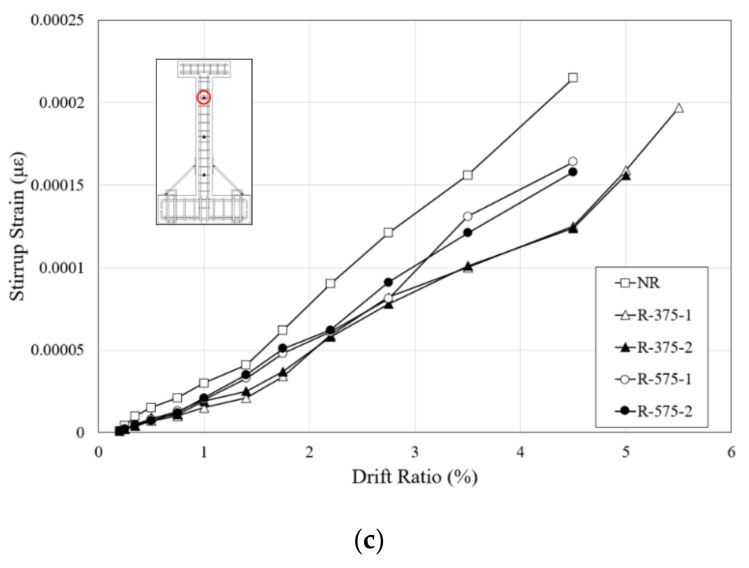
Stirrup strain-drift ratio curves: (**a**) Bottom; (**b**) Middle; (**c**) Top.

Since the experiments on all the specimens were terminated as a result of shear failure, strain on the stirrups showed the shear force applied to the RC columns. Figure 13 shows the stirrup strain-drift ratio relationships of the specimens, and they are divided by the height strain gauges that were attached as aforementioned. With the development of diagonal cracks in the columns, the strain on the stirrups increased. The strain on the stirrup of the NR specimen increased rapidly with the process of loading after the shear cracks occurred, and the stirrup at the bottom of the column yielded before the maximum load. The increase in strain continued as the load was applied. However, the strains on the stirrups of the retrofitted specimens were much smaller than in the NR specimen, which indicates that retrofitting with the RSL system provided strong resistance to lateral load. As the drift ratios of the initial occurrence of the shear cracks and the maximum loads of all the specimens were different, there were various sections with dramatic increases in the stirrup strain. As stress was concentrated in the plastic hinge region of the columns, the lowest stirrups had the greatest strain, the middle stirrups had less, and the lowest strain was measured at the highest stirrup. In the NR specimen, the strain in the stirrups at bottom and central parts of the column increased with the expansion of the width of the shear cracks. On the other hand, the stirrups of the R-375 and R-575 specimens had lower strain than the NR specimen. This means that the RSL system more effectively suppressed the expansion of the cracks developed in shear failure surfaces.

### 4.4. Effective Stiffness

**Figure 14:**
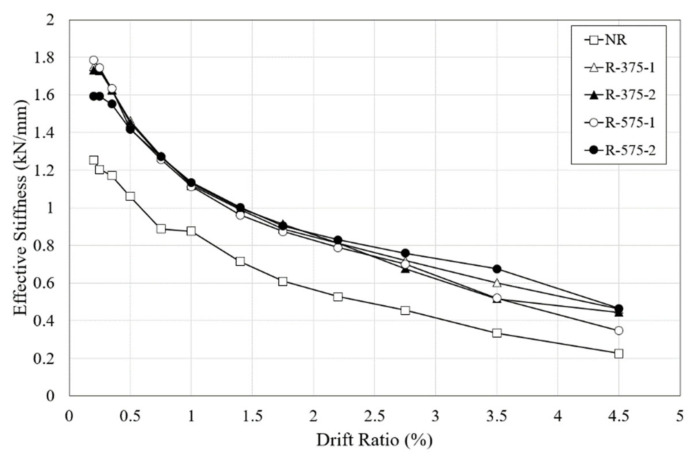
Variation of effective stiffness according to drift ratio.

To evaluate the seismic performance of each specimen based on the test results, effective stiffness was calculated as the slope of the stiffness for each test specimen according to the drift ratio. The variation of the effective stiffness is shown in Figure 14. Stiffness is an important factor to evaluate improved seismic performance because the smaller the decrease in effective stiffness, the longer the stiffness of the specimen is maintained.

For the NR column, the value of the effective stiffness was 1.254 kN/mm at a drift ratio of 0.2%, which was the first drift ratio; this increased to 1.204 kN/mm at a drift ratio of 0.25%, which was the second drift ratio. When the initial flexural cracks occurred, the initial effective stiffness of 1.101 kN/mm decreased by approximately 29% at a drift ratio of 0.75%. Since the effective stiffness at a drift ratio of 0.5% was 1.061 kN/mm, which was only 15% less than the initial effective stiffness, the stiffness of the specimen remained high until just before the initial crack occurred. The sum of the effective stiffness values at all of the drift ratios was 9.32 kN/mm.

The retrofitted specimens generally had higher effective stiffness values than the NR specimen. The initial effective stiffness values of the R-375 and R-575 specimens were found to be about 58% and 53% larger on average, respectively; their respective total effective stiffness values were found to be about 46% and 44% larger on average. Thus, similar to the load-displacement relationships, the length of the retrofit system proved not to be an important factor in retrofitting RC columns.

### 4.5. Energy Dissipation

**Figure 15:**
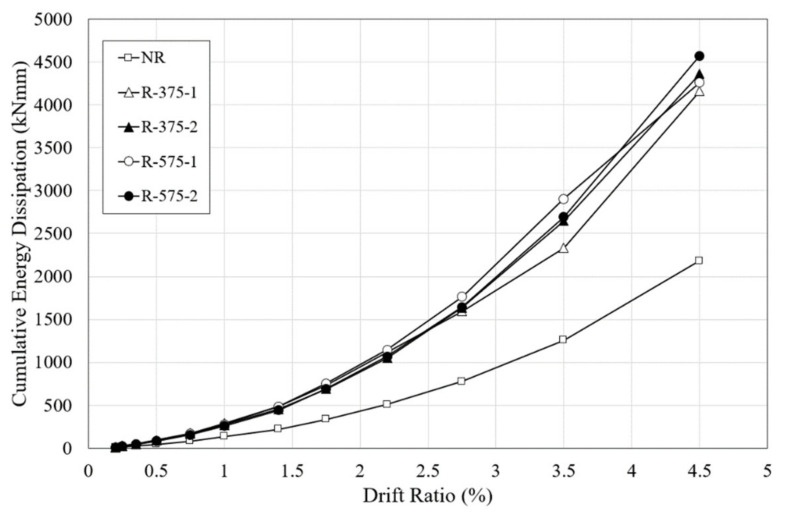
Cumulative energy dissipation capacity according to drift ratio.

The energy dissipation capacity is another important factor when evaluating seismic performance, as well as the ability of a structure to absorb applied seismic energy. The cumulative energy dissipation capacity of all the specimens analyzed here is shown in Figure 15, and is calculated as the area under the load-displacement curve. The cumulative energy dissipation capacity was calculated up to a drift ratio of 4.5%, as experimental assessments of the NR, R-575-1, and R-575-2 specimens were terminated right after the drift ratio of 4.5%. In the initial cycles, at the drift ratio of 0.75% the energy dissipation response of all the specimens was comparable. After the drift ratio of 1%, the retrofitted specimens showed a significantly improved energy dissipation than the un-retrofitted specimen. The R-375 and R-575 specimens showed significant recovery and yielded energy dissipation after 1.5% drift ratio.

It can be observed from the Figure 15, all of the retrofitted specimens dissipate higher energy in each cycle than the NR specimen. The total energy dissipation capacity of the NR specimen was 2181.94 kN·mm, compared with 4158.84 kN·mm in the R-375-1 specimen and 4359.47 kN·mm in the R-375-2 specimen. The average cumulative energy dissipation capacity of the R-375-1 and R-375-2 specimens was 4259.16 kN·mm, which was about 1.95 times that of the NR specimen. The cumulative energy dissipation capacities of the R-575-1 and R-575-2 specimens were 4262.07 kN·mm and 4570.53 kN·mm, respectively. Their average cumulative energy dissipation capacity was 4416.3 kN·mm, which was about 2.02 times of that of the NR specimen. This marked difference in energy dissipation may be attributed to the resistance crack opening or lateral displacement by the RSL system. This clearly proves that the retrofitting effect was noteworthy in both lengths of the RSL system, but the difference in energy dissipation capacity according to the retrofit length was insignificant.

## 5. Conclusions

In this study, a seismic retrofitting method using an RSL system was proposed in order to improve the seismic performance of an RC column. One non-seismically designed RC column and four columns retrofitted with the RSL system were subjected to cyclic lateral loading tests with the retrofitting length as a variable. Crack patterns, load-displacement relationships, effective stiffness, and energy dissipation were evaluated. The following conclusions were drawn:

1. Initial flexural cracks occurred in all of the specimens at the bottom of the column at the drift ratio of 0.75%, but the drift ratios when shear cracks and failure occurred were different. The shear cracks in the NR, R-375, and R-575 specimens were observed at the drift ratios of 1.4%, 2.2%, and 2.75%, respectively. It was demonstrated that the proposed retrofit method was able to make full use of the capacity for deformation afforded by the RSL system. Also, this indicates that longer retrofit length is more effective for suppressing the occurrence of shear cracks;

2. The RSL retrofitting method showed significant improvement in resistance to lateral load. The average maximum load of the R-375 specimens was 2.05 times of that of the NR specimen, and for the R-575 specimens it was 2.04 times of that of the NR specimen. This result suggested retrofitting the plastic hinge enhances the strength of the reinforced concrete columns regardless of the retrofitting length;

3. It was confirmed that the effective stiffness of all the test specimens decreased rapidly after the occurrence of initial cracking. The total effective stiffness values of the R-375 and R-575 specimens were found to be approximately 46% and 44% larger on average, respectively, than the NR specimen. The total effective stiffness of the R-375 specimens was about 2% greater than that of the R-575 specimens, which can be attributed to more effective suppression of torsion because the shorter retrofit was installed closer to the bottom end of the column;

4. The cumulative energy dissipation capacity of the specimens increased rapidly before the initial cracks, but at the drift ratio of 1% the average energy dissipation capacity of all the specimens was only 65.33%. This clarified that the amount of seismic energy dissipated by the specimens increased rapidly due to the occurrence of initial cracks at the drift ratio of 0.75%. This seems to have been due to the rapid decrease in the stiffness of the specimens after the initial cracks occurred, after which the energy dissipation also decreased. Furthermore, the R-375 and R-575 specimens dissipated approximately 2 times more energy, respectively, than the NR specimen. This clearly proves that, while the reinforcing effect was large, the difference in energy dissipation capacity according to the retrofitting length was insignificant.

## Figures and Tables

**Table 1:** Types and variables of the specimens.

Specimen	NR	R-375	R-575	Scheme of Retrofit
Retrofitting techniques	-	RSL	RSL	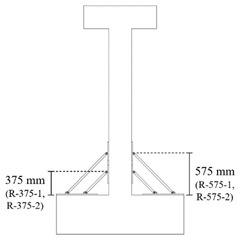
Retrofitted Length	-	375 mm	575 mm	

**Table 2:** Details of the specimens.

Elements	Dimension (mm)	Reinforcement		Section (mm)
		Longitudinal	Transverse (mm)	
Column	250 × 250 × 1750	4-D22	D10@250 mm	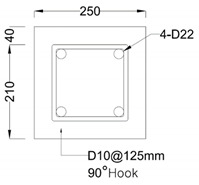
Upper beam	250 × 250 × 800	4-D22	D10@250 mm	
Foundation	1400 × 1270 × 425	10-D22	10-D22	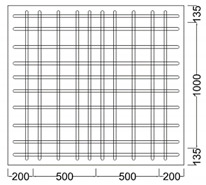

## Data Availability

Data sharing not applicable.
